# Serum levels of immunoglobulin G and M antibodies against SARS-CoV-2 in asymptomatic individuals prior to COVID-19 vaccination in the Democratic Republic of Congo

**DOI:** 10.1371/journal.pone.0343362

**Published:** 2026-03-16

**Authors:** Eric Kasongo Mukenge, Christian Nsimba Lengo, Blaise Matondo-Manzambi Sumbu, Jérémie Masidi Muwonga, Jean-Robert Rissassi Makulo, Mamy Zita Ngole, Ben Izizag Bepouka, Guyguy Kamwiziku, Hippolyte Nani-Tuma Situakibanza, Mireille Nkanga Nganga, Gustave Ntita Ilunga, Fonce Tshibawu Nkunda, Aliocha Natuhoyila Nkodila, Steve Mundeke Ahuka

**Affiliations:** 1 Department of Clinical Biology, University of Kinshasa, Kinshasa, Democratic Republic of Congo; 2 Department of Internal Medicine, University of Kinshasa, Kinshasa, Democratic Republic of Congo; 3 Department of Microbiology, University of Kinshasa, Kinshasa, Democratic Republic of Congo; 4 Department of Family Medicine and Primary Health Care, Protestant University of Congo, Kinshasa, Democratic Republic of Congo; University of the Witwatersrand, SOUTH AFRICA

## Abstract

Despite the production of protective cross-reactive antibodies during COVID-19, data on immunoglobulin G (IgG) and immunoglobulin M (IgM) levels prior to vaccination remain limited in the Democratic Republic of Congo (DRC). This study aimed to assess serum IgG and IgM antibodies against SARS-CoV-2 in asymptomatic individuals COVID-19 vaccination in the DRC. A total of 1,500 individuals who presented for COVID-19 vaccination at the University Clinics of Kinshasa between April 19 and December 31, 2021, were included. The mean age was 47.5 ± 16.0 years, with a predominance of males (69.4%, sex ratio 2:1). Prior to vaccination, 38.8% of participants had elevated IgG levels, while 32.9% had elevated IgM levels. After multivariable adjustment, female sex (adjusted odds ratio [aOR] 1.54; 95% confidence interval [CI] 1.14–2.46), non-healthcare professional status (aOR 2.24; 95% CI 1.42–3.58), and the absence of comorbidities (aOR 2.33; 95% CI 1.38–3.71) remained independently associated with IgM seropositivity. Overall, IgG and IgM seroprevalence were high prior to vaccination. IgM positivity was associated with specific sociodemographic, biochemical, and hematological profiles. These findings highlight the importance of routine serological surveillance before vaccination to better understand infection dynamics and **to** inform public health interventions.

## 1. Introduction

Since its emergence in late 2019, Severe Acute Respiratory Syndrome Coronavirus 2 (SARS-CoV-2) has caused widespread morbidity and mortality worldwide [1]. In sub-Saharan Africa, reported Coronavirus Disease 2019 (COVID-19) case numbers have remained relatively low compared with other regions, despite evidence of substantial undocumented transmission [2]. Limited access to diagnostic testing, high proportions of asymptomatic infections, and inconsistent surveillance systems may contribute to an underestimation of the true burden of infection [3–5].

Serological testing provides a valuable means of assessing prior exposure to SARS-CoV-2 [6,7]. Immunoglobulin M (IgM) antibodies generally indicate recent infection, whereas Immunoglobulin G (IgG) antibodies reflect past exposure and a longer-term humoral response [7,8]. Understanding the distribution of these antibodies before COVID-19 vaccination is essential for interpreting natural immunity, characterizing transmission dynamics, and informing public health decision-making, particularly in resource-constrained settings [9–12].

In the Democratic Republic of the Congo (DRC), few studies have evaluated SARS-CoV-2 antibody levels prior to the rollout of mass vaccination. The extent of silent or undocumented transmission in the population remains unclear. This study aimed to determine the prevalence of anti–SARS-CoV-2 IgG and IgM antibodies among asymptomatic adults presenting for COVID-19 vaccination at a large referral hospital in Kinshasa and to identify factors associated with seropositivity.

## 2. Materials and methods

### 2.1. Study design and population

This cross-sectional analytical study was conducted at the University Clinics of Kinshasa (UCK), Faculty of Medicine, University of Kinshasa in the DRC, between April 19 and December 14, 2021, a period coinciding with the introduction of COVID-19 vaccination in the country. The study population consisted of individuals who voluntarily presented for vaccination against SARS-CoV-2. All participants provided written informed consent or assent before enrollment. Sampling was exhaustive and based on convenience**.** The sample size consisted of 1,500 participants who verbally agreed to participate in the study. Inclusion criteria were being asymptomatic, having accepted blood sampling prior to receiving one of the three available vaccines (AstraZeneca, Moderna, or Pfizer), residing in Kinshasa, and being aged 18 years or older**.** Exclusion criteria included individuals under 18 years of age and patients with COVID-19 who were hospitalized in COVID-19 treatment centers**.**

### 2.2. Data collection and serological analyses

#### 2.2.1. Laboratory procedures.

Blood samples (5 mL) were collected via venipuncture from participants prior to vaccination. Samples were processed and stored at −20°C to −80°C until analysis.

#### 2.2.2. Serological analysis.

Anti–SARS-CoV-2 IgG and IgM antibodies were measured using the Mindray CL-1200i chemiluminescence immunoassay (CLIA) analyzer. This assay detects antibodies binding to SARS-CoV-2 antigens, producing a chemiluminescent signal proportional to the antibody concentration. IgM antibodies indicate recent infection, whereas IgG antibodies reflect prior exposure. Results were interpreted according to manufacturer-defined thresholds (IgG < 10 COI; IgM < 1 COI) [13–15].

#### 2.2.3. Hematological analysis.

Complete blood counts were performed using a BC5150 hematology analyzer, including enumeration of erythrocytes, leukocytes, and platelets, as well as hemoglobin concentration and hematocrit. The neutrophil-to-lymphocyte ratio (NLR) was calculated, with values ≥2 considered indicative of potential COVID-19 infection [16].

#### 2.2.4. Biochemical analysis.

Serum biochemical parameters (urea, creatinine, liver enzymes, bilirubin and CK-MB) were measured using the BS-240Pro automated analyzer, with reference ranges applied according to kit specifications [14].

### 2.3. Statistical analysis

Data were compiled using EpiData software, version 3.1, based on monitoring of the population’s COVID-19 vaccination. The sociodemographic, clinical, and biological characteristics of the participants were compared according to the type of sex (male and female), IgG (IgG < 10 and IgG ≥ 10 COI) and IgM (IgM < 1 and IgM ≥ 1 COI). Continuous variables (such as age, IgG antibodies, IgM antibodies, hematological and biochemical parameters) were expressed as means ± standard deviation (SD) or medians with interquartile ranges (IQR), depending on data distribution. The normality was assessed using the Shapiro-Wilk test. For normally distributed data, comparisons between groups were performed using Student’s *t*-tes*t*. For non-normally distributed data, non-parametric tests, including the Mann-Whitney tests, were used. Qualitative variables (sex, age group, occupation and presence of comorbidities) were presented as absolute numbers and percentages. Group comparisons were performed using the chi-square test. When expected cell counts were small, Fisher’s exact test was applied.

Factors associated with IgM seropositivity were initially evaluated using bivariate logistic regression analyses. Variables with *p* < 0.05 in bivariate analyses were included in the multivariate logistic regression model to control for potential confounders and identify independent predictors of IgM positivity. Adjusted odds ratios (aORs) with 95% confidence intervals (CIs) were reported. Statistical significance was set at *p* < 0.05.

### 2.4. Ethical considerations

The study was conducted in accordance with relevant guidelines and regulations. Ethical approval was obtained from the National Health Ethics Committee (approval No. 201/CNES/BN/PMMF/2021, dated March 28, 2021).

## 3. Results

### 3.1. Description of the study population

The study included 1,500 participants (1,040 males, 460 females) with a mean age of 47.5 ± 16.0 years. No significant difference in mean age was observed between sex (p = 0.631). The distribution of age groups was also similar between males and females (*p* = 0.629).

A significantly higher proportion of males were healthcare professionals compared with females (30.5% vs. 25.5%; *p* = 0.028). Comorbidities were reported in 27.0% of participants, with no significant difference by sex (*p* = 0.087).

Overall, laboratory findings were largely comparable between sex. However, significant differences were observed for several parameters: elevated urea levels were more frequent in males (2.9% vs. 0.9%; *p* = 0.009), elevated alkaline phosphatase levels were more common in females (10.0% vs. 6.0%; *p* = 0.004), low hemoglobin levels were more frequent in males (28.7% vs. 4.6%; *p* < 0.001), and abnormal platelet counts were observed more often in males than in females (15.8% vs. 11.8%; *p* = 0.024) (Table 1).

**Table 1:** Socio-demographic characteristics by sex.

Variable	Over all (n = 1500)	Male (n = 1040)	Female (n = 460)	p
Age, Mean and SD (years)	47.5 ± 16.0	48.0 ± 16.1	46.3 ± 15.9	0.631
Age group				0.629
18 - 39 years	549 (36.6)	373 (35.9)	176 (38.3)	
40 - 59 years	527 (35.2)	368 (35.4)	159 (34.6)	
≥60 years	423 (28.2)	299 (28.8)	124 (27.0)	
Occupation				**0.028**
Healthcare professional	434 (29.0)	317 (30.5)	117 (25.5)	
No Healthcare professional	1065 (71.0)	723 (69.5)	342 (74.5)	
Comorbidity				0.087
No	1095 (73.0)	771 (74.1)	324 (70.6)	
Yes	404 (27.0)	269 (25.9)	135 (29.4)	
Abnormal creatinine	111 (7.4)	72 (6.9)	39 (8.5)	0.167
Abnormal urea	34 (2.3)	30 (2.9)	4 (0.9)	**0.009**
Abnormal Alanine Aminotransferase	224 (14.9)	150 (14.4)	74 (16.1)	0.219
Abnormal Aspartate Aminotransferase	275 (18.3)	193 (18.6)	82 (17.9)	0.405
Abnormal Alkaline Phosphatase	108 (7.2)	62 (6.0)	46 (10.0)	**0.004**
Abnormal Total bilirubin	146 (9.7)	108 (10.4)	38 (8.3)	0.120
Abnormal Creatine Kinase-MB	34 (2.3)	27 (2.6)	7 (1.5)	0.135
Low hemoglobin	319 (21.3)	298 (28.7)	21 (4.6)	**<0.001**
Abnormal platelet	218 (14.5)	164 (15.8)	54 (11.8)	0.024

### 3.2. Hematological and biochemical characteristics of the study population

Among the 1,500 participants, no statistically significant differences were observed between males and females in renal function markers, including creatinine (2.57 vs. 2.55 mg/dL, p = 0.990) and urea (24.0 vs. 24.5 mg/dL, p = 0.779). Liver function tests, including alanine aminotransferase (ALT), aspartate aminotransferase (AST), alkaline phosphatase and total bilirubin, were also comparable between sex (all p > 0.05). Similarly, hematological parameters, including red blood cell count, hemoglobin concentration, hematocrit, platelet count, total white blood cell count, and differential leukocyte counts (neutrophils, lymphocytes, monocytes, and eosinophils), did not differ significantly between males and females (all *p* > 0.05) (Table 2).

**Table 2:** Hematological and biochemical characteristics by sex.

Variable	Over all (n = 1500)	Male (n = 1040)	Female (n = 460)	p
Creatinine (mg/dl)	2.57 (2.5-2.64)	2.57 (2.5-2.7)	2.55 (2.44-2.69)	0.990
Urea (mg/dl)	24.1 (23.6-24.5)	24.0 (23.5-24.4)	24.5 (23.5-25.1)	0.779
Alanine Aminotransferase (UI/L)	19.6 (18.9-20.1)	19.2 (18.2-20.0)	20.5 (19.3-22.0)	0.146
Aspartate Aminotransferase (UI/L)	27.8 (27.0-28.7)	27.5 (26.5-28.6)	28.4 (27.0-29.7)	0.122
Alkaline Phosphatase (UI/L)	57.5 (56.7-58.9)	57.3 (55.9-58.8)	58.0 (55.9-60.3)	0.099
Total Bilirubin (mmol/L)	1.4 (1.2-1.7)	1.3 (1.1-1.7)	1.5 (1.1-5.0)	0.450
Red Blood Cell Count (Cell/mm^3^)	4.67 (4.64-4.71)	4.7 (4.6-4.8)	4.7 (4.6-4.8)	0.568
Hemoglobin (g/L)	13.5 (13.4-13.6)	13.5 (13.3-13.6)	13.6 (13.4-13.8)	0.665
Hematocrit (%)	41.7 ± 11.0	41.6 ± 9.3	42.2 ± 14.2	0.365
Plateletcount (Cell/mm^3^)	218.6 ± 73.2	218.2 ± 75.9	219.4 ± 66.6	0.814
White Blood Cell count (Cell/mm^3^)	5.62 (5.54-5.71)	5.6 (5.5-5.7)	5.58 (5.43-5.75)	0.648
Neutrophils (Cell/mm^3^)	2.48 (2.41-2.53)	2.5 (2.4-2.6)	2.45 (2.34-2.55)	0.907
Lymphocytes (Cell/mm^3^)	2.32 (2.28-2.38)	2.3 (2.2-2.4)	2.29 (2.25-2.38)	0.462
Monocytes (Cell/mm^3^)	0.43 (0.42-0.44)	0.43 (0.41-0.44)	0.43 (0.41-0.44)	0.255
Eosinophils (Cell/mm^3^)	0.19 (0.18-0.20)	0.19 (0.18-0.21)	0.19 (0.16-0.21)	0.311

### 3.3. Study of immunoglobin G (IgG) and immunoglobin M (IgM)

**Fig 1:**
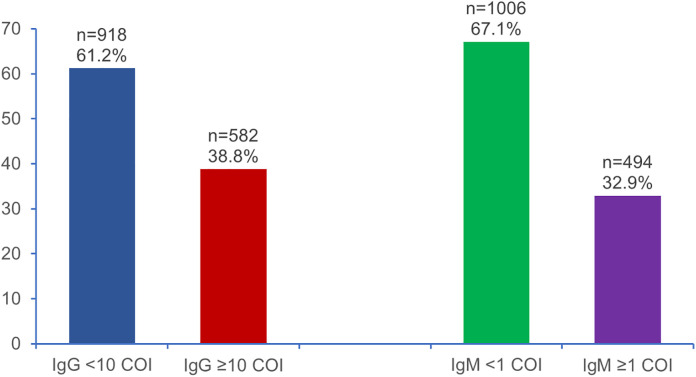
Frequency of positive IgG and IgM levels prior to COVID-19 vaccination.

Fig 1 illustrates the prevalence of positive IgG and IgM antibodies prior to COVID-19 vaccination. Pre-vaccination testing showed that 38.8% of participants had elevated IgG antibodies**,** whereas 32.9% had elevated IgM antibodies (Fig 1 tif).

### 3.4. Comparison of sociodemographic and biological characteristics based on immunoglobulins

Among 1,500 participants, 582 (38.8%) were elevated IgG antibodies (≥10 COI) and 494 (32.9%) were elevated IgM antibodies (≥1 COI). No significant differences were observed between elevated IgG antibodies and normal IgG antibodies participants with respect to age, age group, sex, profession, comorbidity, or most biochemical and hematological markers (all p > 0.05). However, hemoglobin were slightly lower among elevated IgG antibodies individuals (*p* = 0.021). In contrast, elevated IgM antibodies was significantly associated with several variables. It was more frequent among females (*p* = 0.020), non–healthcare workers (*p* = 0.009), and participants without comorbidities (*p* = 0.001). In addition, IgM seropositivity was associated with abnormal alkaline phosphatase levels (*p* = 0.042). Other variables, including age, renal and liver function markers, hemoglobin concentration, and platelet count, were not significantly associated with elevated IgM antibodies (all *p* > 0.05) (Table 3).

**Table 3:** General characteristics of the study population according to IgG and IgM levels.

Variable	IgG			IgM		
	IgG < 10 COI (n = 918)	IgG ≥ 10 COI (n = 582)	p	IgM < 1 COI (n = 1006)	IgM ≥ 1 COI (n = 494)	p
Age, mean and SD (years)	47.4 ± 15.9	47.7 ± 16.1	0.630	46.9 ± 15.7	47.5 ± 16.0	0.723
Age group						0.697
18 - 39 years	338 (36.8)	211 (36.3)		363 (36.1)	186 (37.7)	
40 - 59 years	329 (35.8)	199 (34.2)		353 (35.1)	175 (35.4)	
≥60 years	251 (27.3)	172 (29.6)		290 (28.8)	133 (26.9)	
Sex			0.482			**0.020**
Male	636 (69.3)	404 (69.5)		709 (70.5)	331 (67.1)	
Female	282 (30.7)	177 (30.5)		297 (29.5)	162 (32.9)	
Profession			0.315			**0.009**
Healthcare professional	261 (28.4)	173 (29.7)		306 (30.4)	128 (25.9)	
No Healthcare professional	657 (71.6)	409 (70.3)		700 (69.6)	366 (74.1)	
Comorbidity			0.210			**0.001**
No	678 (73.9)	418 (71.8)		717 (71.3)	379 (76.7)	
Yes	240 (26.1)	164 (28.2)		289 (28.7)	115 (23.3)	
Abnormal creatinine	67 (7.3)	44 (7.6)	0.463	72 (7.2)	39 (7.9)	0.339
Abnormal urea	19 (2.1)	15 (2.6)	0.317	24 (2.4)	10 (2.0)	0.406
Abnormal Alanine Aminotransferase	133 (14.5)	91 (15.6)	0.296	153 (15.2)	71 (14.4)	0.365
Abnormal Aspartate Aminotransferase	169 (18.4)	106 (18.2)	0.490	192 (19.1)	83 (16.8)	0.158
Abnormal Alkaline Phosphatase	69 (7.5)	39 (6.7)	0.313	65 (6.5)	43 (8.7)	**0.042**
Abnormal Total bilirubin	84 (9.2)	62 (10.7)	0.193	106 (10.5)	40 (8.1)	0.079
Abnormal Creatine Kinase-MB	25 (2.7)	9 (1.5)	**0.048**	24 (2.4)	10 (2.0)	0.406
Low hemoglobin	185 (20.2)	134 (23.0)	**0.021**	217 (21.6)	102 (20.6)	0.367
Abnormal platelet	134 (14.6)	84 (14.4)	0.497	150 (14.9)	68 (13.8)	0.305

Abbreviations: COI = Cut-Off Index, IgG = Immunoglobulins G; IgM: Immunoglobulins M; SD: Standard deviation

Median IgG antibodies were significantly higher among elevated IgG antibodies participants (17.2 COI) than among normal IgG antibodies participants (3.2 COI; *p* < 0.001), whereas IgM antibodies did not differ significantly according to IgG status (*p* = 0.161). Conversely, elevated IgM antibodies participants exhibited markedly higher IgM antibodies (1.5 COI) compared with IgM-seronegative participants (0.7 COI; *p* < 0.001), while IgG antibodies were similar across IgM status groups (*p* = 0.335). No significant differences were observed in renal function markers (creatinine and urea) or liver function markers (ALT, AST, and total bilirubin) between IgG- or IgM-seropositive and -seronegative participants (all *p* > 0.05). Hematological parameters, including red blood cell count, hemoglobin concentration, hematocrit, platelet count, total white blood cell count, and neutrophil and lymphocyte counts, were largely comparable across IgG and IgM groups. However, slight but statistically significant differences by IgG status were observed for monocyte and eosinophil counts (monocytes: 0.41 vs. 0.43 × 10³/mm³, *p* = 0.023; eosinophils: 0.19 vs. 0.20 × 10³/mm³, *p* = 0.020). No hematological parameter differed significantly according to IgM status (Table 4).

**Table 4:** Biological characteristics of the study population according to IgG and IgM levels.

Variable	IgG			IgM		
IgG < 10 COI (n = 918)	IgG ≥ 10 COI (n = 582)	p	IgM < 1 COI (n = 1006)	IgM ≥ 1 COI (n = 494)	p
IgG (COI)	3.2 (2.8-3.6)	17.2 (16.7-17.9)	**<0.001**	6.15 (5.30-7.30)	7.2 (6.45-7.70)	0.335
IgM (COI)	0.7 (0.6-0.7)	0.7 (0.6-0.8)	0.161	0.70 (0.60-0.70)	1.5 (1.5-1.6)	**<0.001**
Creatinine (mg/dl)	2.55 (2.48-2.65)	2.57 (2.48-2.71)	0.862	2.50 (2.37-2.65)	2.57 (2.51-2.63)	0.596
Urea (mg/dl)	24.21 (23.51-24.68)	23.9 (23.4-24.4)	0.554	23.86 (23.12-24.59)	24.1 (23.6-24.4)	0.975
ALT (UI/L)	19.8 (18.8-20.8)	19.3 (18.2-20.3)	0.694	19.60 (18.53-20.65)	19.6 (18.8-20.1)	0.645
AST (UI/L)	28.1 (27.1-29.5)	27.2 (26.3-28.5)	0.951	26.92 (26.05-28.50)	27.7 (27.0-28.7)	0.257
Total bilirubin (mmol/L)	1.7 (1.3-2.2)	1.1 (0.9-1.3)	0.453	1.3 (1.0-2.5)	1.35 (1.20-1.70)	0.312
RBCC (Cell/mm^3^)	4.69 (4.63-4.74)	4.65 (4.61-4.71)	0.425	4.69 (4.63-4.77)	4.67 (4.64-4.71)	0.618
Hemoglobin (g/L)	13.5 (13.4-13.6)	13.4 (13.3-13.6)	0.450	13.5 (13.3-13.6)	13.5 (13.4-13.6)	0.556
Hematocrit (%)	41.7 ± 9.4	41.9 ± 13.1	0.701	41.7 ± 11.6	41.8 ± 11.0	0.832
Plateletcount (Cell/mm^3^)	220.8 ± 75.4	214.9 ± 69.6	0.145	218.1 ± 68.9	218.5 ± 73.2	0.899
WBCC (Cell/mm^3^)	5.70 (5.59-5.85)	5.51 (5.38-5.68)	0.682	5.67 (5.51-5.85)	5.61 (5.55-5.71)	0.861
Neutrophils (Cell/mm^3^)	2.49 (2.41-2.56)	2.45 (2.36-2.59)	0.349	2.51 (2.35-2.62)	2.49 (2.41-2.54)	0.829
Lymphocytes (Cell/mm^3^)	2.37 (2.30-2.45)	2.26 (2.20-2.33)	0.524	2.37 (2.27-2.46)	2.32 (2.28-2.37)	0.984
Monocytes (Cell/mm^3^)	0.43 (0.42-0.45)	0.41 (0.40-0.43)	**0.023**	0.43 (0.41-0.44)	0.43 (0.42-0.44)	0.484
Eosinophils (Cell/mm^3^)	0.20 (0.18-0.21)	0.19 (0.17-0.21)	**0.020**	0.19 (0.17-0.22)	0.19 (0.18-0.20)	0.745

Abbreviations: COI = Cut-Off Index, IgG = Immunoglobulines G; IgM: Immunoglobulines M, ALT: Alanine Aminotransferase; AST: Aspartate Aminotransferase; RBCC: Red Blood Cell Count; WBCC: White Blood Cell count.

### 3.5. Determinants of positive IgM levels in vaccinated individuals

In univariate analysis, female sex, non-healthcare occupation, absence of comorbidities, and abnormal Alkaline Phosphatase (ALP) were significantly associated with IgM seropositivity (all p < 0.05). Specifically, females had higher odds of IgM positivity compared with males (cOR 2.84, 95% CI 1.93–4.47, p = 0.019), non-healthcare professionals had higher odds than healthcare professionals (cOR 3.25, 95% CI 1.98–4.59, p = 0.002), and participants without comorbidities had higher odds than those with comorbidities (cOR 3.33, 95% CI 1.19–4.71, p = 0.003). Abnormal ALP was also associated with increased odds (cOR 2.38, 95% CI 1.92–3.06, p = 0.012). After adjustment in multivariate logistic regression, female sex (aOR 1.54, 95% CI 1.14–2.46, p = 0.023), non-healthcare occupation (aOR 2.24, 95% CI 1.42–3.58, p = 0.012), and absence of comorbidities (aOR 2.33, 95% CI 1.38–3.71, p = 0.024) remained significantly associated with IgM seropositivity (Table 5).

**Table 5:** Determinants of positive IgM levels in vaccinated individuals.

Variable	Univariate analysis		Multivariate analysis	
p	cOR (95%CI)	p	aOR(95%CI)
Gender				
Male		1		1
Female	**0.019**	2.84 (1.93-4.47)	**0.023**	1.54 (1.14-2.46)
Occupation				
Healthcare professional		1		1
No Healthcare professional	**0.002**	3.25 (1.98-4.59)	**0.012**	2.24 (1.42-3.58)
Comorbidity				
Yes		1		1
No	**0.003**	3.33 (1.190-4.71)	**0.024**	2.33 (1.38-3.71)
Alkaline Phosphatase				
Normal		1		1
Abnormal	**0.012**	2.38 (1.92-3.06)	0.118	1.38 (0.92-2.07)

Abbreviation: cOR = Crud Odd Ratio; aOR: Ajusted Odd Ratio; CI: Confiance Interval, P represents the value of

## 4. Discussion

Given the low COVID-19 vaccination coverage in the DRC and the substantial epidemiological burden of SARS-CoV-2 in the community, understanding population-level IgM and IgG immune response is essential. This study aimed to assess the seroprevalence of IgG and IgM antibodies against SARS-CoV-2 among asymptomatic individuals as a baseline prior to the implementation of COVID-19 vaccination. Our findings indicate that 38.8% of participants were IgG-seropositive, while 32.9% exhibited elevated IgM levels before vaccination. These results suggest that a considerable proportion of the population had been previously exposed to SARS-CoV-2, either through symptomatic or asymptomatic infection. The detection of IgG antibodies reflects the establishment of a humoral immune response following prior infection or exposure [17]. Although IgM responses are generally transient, IgM seropositivity is commonly interpreted as a marker of recent or ongoing infection [18]. The concomitant presence of IgG and IgM antibodies may therefore reflect different stages of post-infection immune evolution [19].

The relatively high seroprevalence observed among unvaccinated individuals indicates widespread viral circulation prior to the vaccination campaign. This finding highlights the contribution of undetected infections to community transmission, particularly in resource-limited settings with restricted access to diagnostic testing. These results underscore the importance of pre-vaccination serological screening to document pre-existing immunity and to more accurately evaluate the impact of vaccination on population-level immunity. However, interpretation should account for interindividual variability in immune responses, antibody persistence, and assay performance. The presence of pre-existing immunity also has important implications for vaccination strategies. Individuals who are IgG-seropositive prior to vaccination may require only a single vaccine dose rather than a two-dose regimen, as suggested by several studies and public health agencies [20]. Knowledge of baseline immune status may facilitate more efficient vaccine allocation by prioritizing immunologically naïve individuals (IgG- and IgM-negative) and may also assist in monitoring the risk of exaggerated immune responses in those with high baseline antibody levels. Comparative analyses showed that participants with positive IgG status had higher frequencies of CK-MB abnormalities, lower hemoglobin (Hb) levels, and higher median monocyte and eosinophil counts. These findings suggest that some individuals may retain biological sequelae following SARS-CoV-2 infection, even in the absence of clinical symptoms [21]. Elevated CK-MB levels may reflect mild or transient myocardial injury, which has been reported in post-COVID-19 patients, including those who were not hospitalized [22]. Reduced Hb levels may be attributable to chronic inflammation or post-infectious bone marrow suppression [23]. In addition, moderate monocytosis and eosinophilia may represent residual immune activation or adaptive post-exposure immune responses [24], potentially associated with specific immunological profiles in individuals mounting detectable humoral response [25].

Multivariate analysis identified female sex (aOR 1.54; 95% CI 1.14–2.46), non-healthcare professional status (aOR 2.24; 95% CI 1.42–3.58), and absence of comorbidities (aOR 2.33; 95% CI 1.38–3.71) as independent predictors of IgM seropositivity. These findings suggest that certain demographic and clinical characteristics may be associated with recent or ongoing SARS-CoV-2 infection, even among asymptomatic individuals. The higher prevalence of IgM positivity observed in women may reflect a more rapid or robust humoral immune response [26], although behavioral factors such as differential exposure risk or healthcare-seeking behaviors may also play a role [27]. The increased IgM seroprevalence among non-healthcare professionals likely reflects greater community exposure compared with healthcare workers, who typically adhere to stricter infection prevention measures. Interestingly, the association between IgM seropositivity and the absence of comorbidities may be explained by a greater capacity to mount a detectable immune response or by the occurrence of mild, transient infections compatible with isolated IgM detection.

Overall, these findings reinforce the value of pre-vaccination serological assessment for documenting pre-existing immunity, particularly among asymptomatic individuals. They also highlight the need for a more nuanced understanding of immune response profiles according to sex, occupational exposure, and health status. Nevertheless, these observations should be interpreted with caution, as longitudinal follow-up studies are required to assess the temporal dynamics and clinical relevance of these immune and biological alterations. Further research is warranted to determine whether these changes represent transient post-infectious phenomena or have longer-term clinical implications.

## 5. Conclusion

This study highlights the significant presence of a humoral immune response (IgG and IgM positive) in a significant proportion of asymptomatic individuals prior to vaccination against COVID-19. These results suggest the existence of previous undocumented exposure to SARS-CoV-2, accompanied by a measurable immune response, even in the absence of symptoms. From a public health perspective, these data underscore the importance of better understanding the determinants of natural immunity in populations targeted by vaccination campaigns. Identifying pre-existing immunological profiles could help guide vaccination strategies, particularly by optimizing the schedule or the need for booster shots for certain group. Future research incorporating longitudinal analysis would be needed to assess the durability of this pre-vaccination immunity and its protective role against subsequent forms of the disease.

## Supporting information

S1 Dataset Database of sociodemographic and biological characteristics. (XLSX)
